# SUV39H2 methylates and stabilizes LSD1 by inhibiting polyubiquitination in human cancer cells

**DOI:** 10.18632/oncotarget.4760

**Published:** 2015-07-03

**Authors:** Lianhua Piao, Takehiro Suzuki, Naoshi Dohmae, Yusuke Nakamura, Ryuji Hamamoto

**Affiliations:** ^1^ Section of Hematology/Oncology, Department of Medicine, The University of Chicago, Chicago, IL, USA; ^2^ Biomolecular Characterizaion Unit, RIKEN Center for Sustainable Resource Science, Wako, Saitama, Japan

**Keywords:** SUV39H2, carcinogenesis, LSD1, non-histone protein methylation

## Abstract

LSD1 is a histone lysine demethylase, which is highly expressed in multiple types of human cancer. Although its roles in transcriptional regulation have been well-studied, functional regulation of LSD1 by post-translational modifications still remains unknown. Here, we demonstrate that the histone lysine methyltransferase SUV39H2 trimethylated LSD1 on lysine 322. Knockdown of SUV39H2 resulted in a decrease of LSD1 protein even though the mRNA levels were unchanged. SUV39H2-induced LSD1 methylation suppresses LSD1 polyubiquitination and subsequent degradation. In addition, we also observed indirect effect of SUV39H2 overexpression on LSD1-target genes. Our results reveal the regulatory mechanism of LSD1 protein through its lysine methylation by SUV39H2 in human cancer cells.

## INTRODUCTION

LSD1 (also known as KDM1A, AOF2, and BHC110), the first-identified histone lysine-specific demethylase [1], is an amine oxidase that catalyzes lysine demethylation in a flavin adenine dinucleotide (FAD)-dependent oxidative reaction. LSD1 is composed of three domains; a SWIRM domain that is a conserved motif shared by many chromatin regulatory complexes, an amine oxidase (AO) domain and a Tower domain [2-4]. LSD1 is involved in both transcriptional gene repression and activation through its demethylation of H3K4 and H3K9 [1, 5]. LSD1 can only demethylate mono- and dimethyl lysine residues, and not trimethyl lysine residues, because it requires a lone pair of electrons only present on mono- and dimethylated lysine residues [6]. It was also reported that LSD1 demethylates lysine residues at non-histone proteins including p53, MYPT1, DNMT1 and E2F1 [7-10]. LSD1 demethylates p53 at lysine 370 and represses p53-mediated transcriptional upregulation including induction of apoptosis [8, 11, 12]. Subsequently LSD1 was found as a member of multiple complexes including a CoREST complex and a NuRD complex, both of which function in transcription repression [13-15]. In addition, overexpression of LSD1 was detected in various types of human cancer, and high levels of LSD1 were correlated with poor outcome of cancer patients [16-18]. Concordantly, depletion of LSD1 inhibits cancer cell proliferation [16]. These results suggested that LSD1 could be a promising therapeutic target for development of drugs to treat cancers. Indeed, clinical trials of LSD1 inhibitors have just started for small cell lung carcinoma and acute myeloid leukemia [6]. With regard to the post-translational modifications of LSD1, Nam *et al*. reported that protein kinase Cα (PKCα) phosphorylates LSD1 and plays a critical role in rhythmicity and phase resetting of the circadian clock [19]. However, other modifications influencing functions of LSD1 have not been elucidated, so far.

Suppressor of Variegation 3-9 Homologue 2 (SUV39H2, also known as KMT1B) is a histone lysine-specific methyltransferase that was firstly reported to methylate lysine 9 of histone H3 (H3K9) [20]. In general, histone H3K9 methylation is involved in heterochromatin formation and transcriptional repression. Genetic ablation in the *SUV39H1* and *SUV39H2* genes result in severe chromosomal instabilities, such as abnormally-long telomeres with reduced binding of chromobox proteins Cbx1, Cbx3 and Cbx5 [21, 22]. We recently reported that SUV39H2 methylates histone H2AX and regulates the DNA repair pathway through regulation of γ-H2AX activity in human cancer [23]. Since the expression of SUV39H2 is restricted in testis in adult tissues and is significantly elevated in various cancer types such as non-small cell lung cancer, bladder cancer and prostate cancer [23], SUV39H2 appears to be an ideal target for development of anti-cancer treatment.

In the present study, we demonstrate that SUV39H2 trimethylates LSD1 on lysine 322. SUV39H2-mediated LSD1 methylation inhibits polyubiquitination, which leads to stabilization of the LSD1 protein. Our studies unveil a novel mechanism of SUV39H2 in human cancer through the lysine methylation of LSD1.

## RESULTS

### SUV39H2 methylates lysine 322 on LSD1 both *in vitro* and *in vivo*

**Figure 1:**
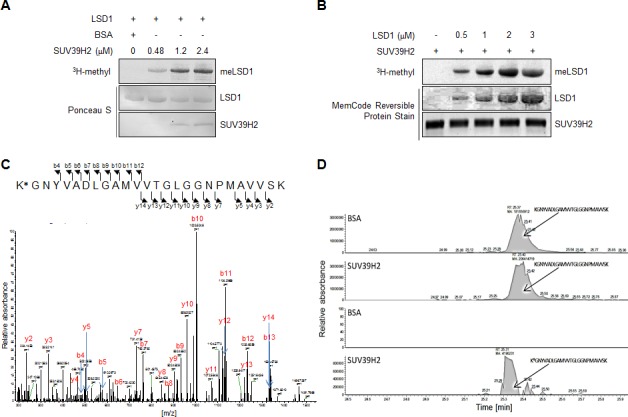
SUV39H2 methylates LSD1 **A.** Recombinant LSD1 protein was methylated by SUV39H2 in a dose-dependent manner. An *in vitro* methyltransferase assay was performed by using purified His-tagged LSD1 and different amount of SUV39H2 recombinant proteins. Methylated LSD1 was detected by fluorography. Amounts of loading proteins were evaluated by staining with Ponceau S. **B.** Confirmation of the *in vitro* methyltransferase assay. Different amount of LSD1 protein was mixed with SUV39H2 in the presence of S-adenosyl-L-[methyl-^3^H]-methionine. Methylated LSD1 was detected by fluorography. Amounts of loading proteins were evaluated by staining with MemCode^TM^ Reversible Protein Stain (Thermo Fisher Scientific). **C.** The MS-MS spectrum corresponding to the trimethylated LSD1 322–347 peptide. The 42 Da increase of the Lys 322 residue was observed. **D.** MS chromatograms of unmodified and trimethylated LSD1 322–347 peptides.

**Figure 2:**
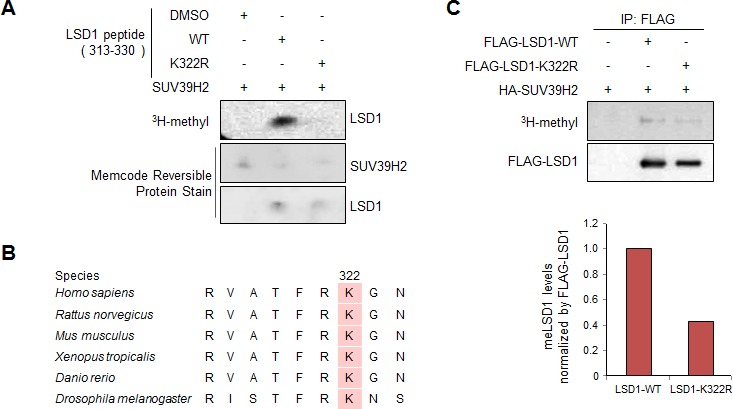
Lys 322 on LSD1 methylation by SUV39H2 both *in vitro* and *in vivo* **A.** *In vitro* methyltransferase assay indicated that LSD1 peptide (amino acid residues 313-330) was methylated by SUV39H2 but not Lys 322-substituted LSD1 peptide (K322R). Amounts of loading proteins were evaluated by staining the MemCode^TM^ Reversible Protein Stain (Thermo Fisher Scientific). **B.** Amino acid sequence indicated that the methylation site Lys 322 was highly conserved across species. **C.** Methylation of LSD1 in human cells was confirmed by *in vivo* labeling experiment. 293T cells were transfected with FLAG-LSD1-WT or FLAG-LSD1-K322R in the presence of HA-SUV39H2 and treated with methionine-free medium, including cycloheximide and chloramphenicol. They were then labeled with L-[methyl-^3^H] methionine for 3 hours. Cell lysates were immunoprecipitated with FLAG-M2 agarose, and methylated LSD1 was visualized by fluorography. The membrane was immunoblotted with an anti-FLAG (an internal control) antibody.

We performed an *in vitro* methyltransferase assay using recombinant LSD1 protein with a variety of recombinant histone methyltransferases to identify an enzyme(s) that would possibly methylate LSD1 and found that the histone methyltransferase SUV39H2 could methylate LSD1 in a dose-dependent manner (Figure 1A, 1B and Supplementary Figure S1). Subsequently, we applied liquid chromatography-tandem mass spectrometry (LC-MS/MS) and identified that lysine 322 on LSD1 was trimethylated by SUV39H2 (Figure 1C and 1D). To further confirm this methylation site, we synthesized the wild-type peptide covering amino acids 313-330 of LSD1 (WT) and the Lys 322-substituted LSD1 peptide (K322R), and performed an *in vitro* methyltransferase assay. Consequently, we detected a strong methylation signal only in the wild-type LSD1 peptide but not the substituted LSD1 peptide (K322R) (Figure 2A). In addition, lysine 322 of LSD1, the methylation site, is highly conserved across species (Figure 2B), supporting that this lysine methylation might have a critical role in the function of LSD1. Furthermore, we validated the methylation of LSD1 by SUV39H2 in 293T cells that were transfected with a FLAG-LSD1 wild-type (WT) vector or a FLAG-LSD1-K322R vector together with an HA-SUV39H2. *In vivo* labeling experiments revealed a strong signal corresponding to methylated LSD1 in FLAG-LSD1-WT-transfected cells, but the specific signal was significantly diminished in FLAG-LSD1-K322R-tranfected cells (Figure 2C). Taken together, these results imply that SUV39H2 methylates lysine 322 on LSD1 both *in vitro* and *in vivo*.

### SUV39H2 stabilizes LSD1 via inhibiting polyubiquitination

**Figure 3:**
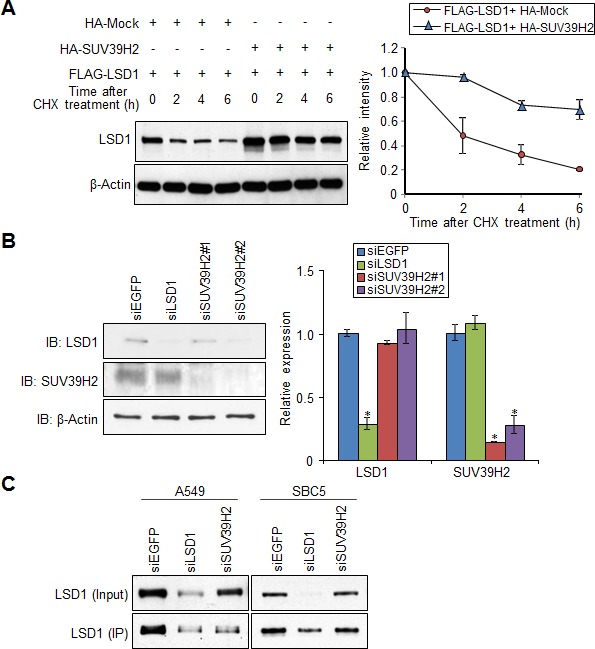
SUV39H2 stabilizes LSD1 protein **A.** FLAG-LSD1 was co-expressed with HA-Mock or HA-SUV39H2 into 293T cells. After treating cells with cycloheximide (CHX) (100 μg/ml) for indicated time intervals, expression of LSD1 was examined (left panel). The intensity of LSD1 protein for each time point was quantified by densitometry and plotted (right panel). Results are the mean ± SD of three independent experiments. **B.** A549 cells were transfected with control EGFP, LSD1 and two different SUV39H2 siRNAs. Expression of LSD1, SUV30H2 and β-Actin (internal control) was examined by western blot (left panel). The mRNA levels of *LSD1* and *SUV39H2* were quantified by real-time PCR. All error bars indicate SEM of two independent experiments. *P*-values were calculated using Student's *t*-test (*P < 0.05). **C.** Lysates from A549 and SBC5 cells transfected with control EGFP, LSD1 and SUV39H2 siRNA were immunoprecipitated with an anti-LSD1 antibody. LSD1 protein levels were examined by western blot analysis.

We previously reported that biological effects of lysine methylation are categorized into 5 classes, and one of them is to regulate the stability of substrate protein [6]. To examine whether SUV39H2-mediated methylation affects protein stability of LSD1, we co-expressed FLAG-tagged LSD1 with Mock vector or HA-tagged SUV39H2 in 293T cells. The cells were treated with cycloheximide (CHX) to block new protein synthesis. We found that LSD1 protein degraded much more rapidly in the cells co-transfected with a control mock vector, compared to those co-transfected with HA-tagged SUV39H2 (Figure 3A), indicating that LSD1 proteins were degraded much faster probably due to the lack of its methylation by SUV39H2. Consistently, we detected a remarkable reduction of LSD1 protein levels when knocking down endogenous expression of SUV39H2 in A549 cells even though mRNA levels of *LSD1* were unchanged (Figure 3B). In addition, when LSD1 was immunoprecipitated with an anti-LSD1 antibody from whole cell extracts of A549 or SBC5 cells treated with siEGFP, siLSD1 or siSUV39H2, a significant decrease of LSD1 protein was also observed in the immunoprecipitants after treatment with siSUV39H2 as well as siLSD1 (Figure 3C). Hence, these results indicate that SUV39H2 appears to stabilize LSD1 protein via methylation.

**Figure 4:**
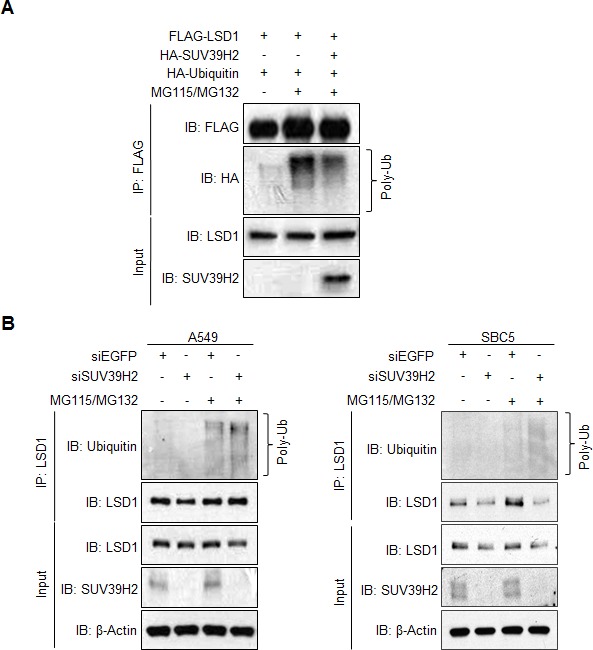
SUV39H2-dependent LSD1 methylation inhibits LSD1 protein degradation mediated by polyubiquitination **A.** FLAG-LSD1 and HA-Ubiquitin were co-expressed in 293T cells with or without HA-SUV39H2. Cells were incubated with 5 μM MG115 and 10 μM MG132 for 6 hours before lysis. LSD1 protein was immunoprecipitated with anti-FLAG M2 agarose beads, and polyubiquitinated LSD1 proteins were detected by anti-HA antibody. **B.** After incubation with siEGFP or siSUV39H2 for 72 hours, A549 (left panel) or SBC5 (right panel) cells were treated with 5 μM MG115 and 10 μM MG132 for 6 hours. Cell lysates were immunoprecipitated with anti-LSD1 antibody. Polyubiquitinated LSD1 protein was detected using an anti-Ubiquitin antibody.

Protein polyubiquitination is known as a signal for protein degradation. We hypothesized that SUV39H2 could stabilize LSD1 protein through inhibiting polyubiquitination of LSD1, and co-expressed FLAG-LSD1 and HA-Ubiquitin with HA-Mock or HA-SUV39H2 in 293T cells. Ubiquitinated LSD1 was observed in the FLAG-LSD1 immunoprecipitants purified from the cells treated with MG115 and MG132. We observed remarkable reduction of ubiquitinated LSD1 in the LSD1 immunoprecipitants purified from the cells co-expressing with SUV39H2, indicating that SUV39H2 seems to reduce polyubiquitination levels of LSD1 protein (Figure 4A). Furthermore, endogenous ubiquitination levels of LSD1 protein were examined by knocking down the expression of SUV39H2. Consistently, attenuation of SUV39H2 expression resulted in an increase of polyubiquitination levels on LSD1 in A549 and SBC5 cancer cells (Figure 4B). These results imply that SUV39H2 stabilizes LSD1 through inhibiting polyubiquitination levels of LSD1.

### SUV39H2-mediated lysine 322 methylation regulates LSD1-downstream genes

**Figure 5:**
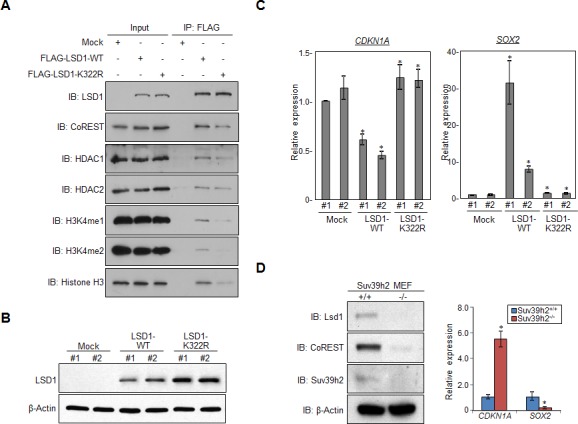
Methylation of LSD1 at lysine 322 is critical for its binding to CoREST, and regulating targeted genes **A.** 293T cells were individually transfected with FLAG-Mock, FLAG-LSD1 or FLAG-LSD1mutant (K322R) vectors, and cell extracts were immunoprecipitated with anti-FLAG M2 agarose beads. **B.** Expression levels of LSD1 proteins were examined in stably expressing wild-type LSD1 or mutant LSD1 (K322R) HeLa cells. **C.** The mRNA levels of *CDKN1A* and *SOX2* were quantified by real-time PCR. All error bars indicate SEM of four independent experiments. *P*-values were calculated using Student's *t*-test (*P < 0.05). **D.** Expression levels of Lsd1 and CoREST proteins from Suv39h2 wild-type (Suv39h2^+/+^) and Suv39h2 null (Suv39h2^−/−^) MEF cells were examined by western blot (left panel). The mRNA levels of *CDKN1A* and *SOX2* were quantified by real-time PCR. All error bars indicate SEM of two independent experiments. *P*-values were calculated using Student's *t*-test (*P < 0.05).

According to our previous report, lysine methylation also regulates protein-protein interactions [6]. LSD1 is a part of the CoREST complex, and lysine 322, the methylation site by SUV39H2, is close to the portion of LSD1 that was reported to be critical for the interaction between LSD1 and CoREST based on the structural analysis [24]. Therefore, we investigated whether SUV39H2-dependent LSD1 methylation on lysine 322 affects the interaction between LSD1 and CoREST. As shown in Figure 5A, we examined binding affinities of wild-type LSD1 (LSD1-WT) and K322R mutant-type LSD1 (LSD1-K332R) to CoREST, and interestingly, LSD1-K332R showed a significant reduction of binding affinities to CoREST compared to LSD1-WT. Moreover, K322R mutant of LSD1 caused lower binding affinities to H3K4me1, H3K4me2 and total histone H3 (Figure 5A). Taken together, SUV39H2-mediated methylation of LSD1 at lysine 322 was important for its binding affinities to CoREST.

**Figure 6:**
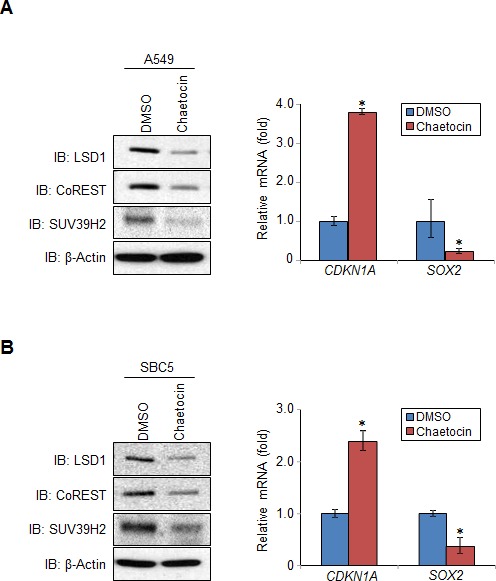
Methylation of LSD1 at lysine 322 affects LSD1 regulation of targeted genes Chaetocin, an inhibitor of the SU(VAR)3-9 was treated into A549 cells **A.** and SBC5 cells **B.** at the dose of 0.5 μM for 24 hours. The expression levels of LSD1 and CoREST proteins were examined by western blot (left panel). The mRNA levels of *CDKN1A* and *SOX2* were quantified by real-time PCR (right panel). All error bars indicate SEM of two independent experiments. *P*-values were calculated using Student's *t*-test (*P < 0.05).

LSD1 is an essential epigenetic regulator, and critical in regulating expression of several downstream target genes [25]. To examine whether SUV39H2-medidated methylation has an influence on transcriptional regulation of LSD1-target genes, we established stable HeLa cell clones that constitutively overexpress LSD1-WT or LSD1-K322R (Figure 5B). We then examined expression levels of *CDKN1A* (*p21/CIP1*) and *SOX2*, which are LSD1-target genes [26, 27], and found that LSD1-WT overexpressing cells showed lower *CDKN1A* expression and higher *SOX2* expression compared to control Mock cells. However, we couldn't observe expression changes of these genes in LSD1-K322R overexpressing cells (Figure 5C). Consistent with these results, LSD1-K322R overexpressing cells showed lower growth rate than LSD1-WT overexpressing cells (Supplementary Figure S2). Next, we examined expression of *Cdkn1a* and *Sox2* in *Suv39h2*-null (*Suv39h2*^−/−^) mouse embryonic fibroblast (MEF) cells established from *Suv39h2* knockout mouse [21]. Compared to wild-type MEFs, we observed diminished protein expressions of Lsd1 and CoREST in *Suv39h2*^−/−^ MEFs (Figure 5D). Consistently, we found increased *Cdkn1a* and reduced *Sox2* mRNA levels in *Suv39h2*^−/−^ MEFs compared to wild-type MEFs (Figure 5D). Similar results were also observed in the cells treated with chaetocin, an inhibitor of the SU(VAR)3-9 [28, 29] (Figure 6A and 6B).

## DISCUSSION

In the present study, we identified that LSD1 was trimethylated by SUV39H2 at lysine 322, and that SUV39H2-mediated LSD1 methylation stabilized the protein levels of LSD1 through inhibition of its polyubiquitination. Overexpression of SUV39H2 attenuated LSD1 polyubiquitination, whereas depletion of SUV39H2 enhanced LSD1 polyubiquitination and enhanced degradation of LSD1, indicating that SUV39H2-induced LSD1 methylation impedes LSD1 polyubiquitination. In addition, the methylation of lysine 322 on LSD1 by SUV39H2, was critical for the interaction of LSD1 with CoREST. Moreover, since this SUV39H2-mediated LSD1 methylation enhanced the binding affinity to histone H3, we hypothesized that methylation could possibly influence transcriptional regulation of LSD1-target genes. Indeed, we confirmed that LSD1 methylation at lysine 322 played critical roles in the regulation of LSD1-target genes, *CDKN1A* and *SOX2*, whose dysregulations are involved in human tumorigenesis [30, 31]. Among biological functions of protein lysine methylation in human cancer we previously categorized [6], this study implies that SUV39H2-mediated LSD1 methylation may increase the stability of LSD1 proteins by suppressing polyubiquitination, and may also alter LSD1-CoREST interaction, which causes aberrant transcriptional regulation of LSD1-downstream genes.

Many reports have indicated that dysregulation of protein lysine methyltransferases and protein lysine demethylases has important roles in human tumorigenesis [32-37]. LSD1 is highly expressed in multiple types of cancer, including bladder cancer, oestrogen-receptor-negative breast cancer, colorectal cancer, lung cancer and prostate cancer [16, 17, 38]. Previous data have also indicated that LSD1 functioned as an essential regulator of leukemia stem cell (LSC) potential [39], and its inhibition could reactivate the all-trans-retinoic acid differentiation pathway in acute myeloid leukemia [40]. As mentioned above, we previously reported that overexpression of SUV39H2 in various cancer types such as non-small cell lung cancer, bladder cancer and prostate cancer [23]. These accumulated data indicate that both SUV39H2 and LSD1 could be promising candidates for anti-cancer drug development. Several results have shown that LSD1 inhibitor could suppress different types of cancer [41, 42]. In particular, a Phase I clinical trial of GSK2879552, an LSD1-specific inhibitor, has been initiated for patients with relapsed/refractory small cell lung cancer, and a Phase I clinical trial of the novel LSD1 inhibitor ORY-1001 has begun for acute myeloid leukaemia [6]. Combination therapy involving LSD1 inhibitors could also be a viable approach, such as HDACs inhibitors and ATRA inhibitors. In fact, inhibition of LSD1 could sensitize cancer cells to HDAC inhibitors [42, 43]. Our study indicated a high correlation between SUV39H2 and LSD1, which imply a new viable approach combining LSD1 and SUV39H2 inhibitors in cancer therapy.

## MATERIALS AND METHODS

### Cell culture

293T, HeLa and A549 cells were from American Type Culture Collection (ATCC) in 2001 and 2003, and tested and authenticated by DNA profiling for polymorphic short tandem repeat (STR) markers (Supplementary Table S1). SBC-5 cells were from the Japanese Collection of Research Bioresources in 2001, and were tested and authenticated by DNA profiling for polymorphic short tandem-repeat markers (Supplementary Table S1). Both cell lines were grown in monolayers in appropriate media: Dulbecco's modified Eagle's medium (D-MEM) for 293T cells; RPMI1640 medium for A549; Eagle's Minimum Essential Medium (E-MEM) for HeLa and SBC-5 cells supplemented with 10% fetal bovine serum and 1% antibiotic/antimycotic solution (Sigma-Aldrich, St. Louis, MO). We also generated stable HeLa cell lines constitutively expressing LSD1. The pCAGGS-LSD1-3xFLAG or empty pCAGGS-3xFLAG mock vector was transfected into HeLa cells by FuGENE6 (Roche Applied Science, Penzberg, Germany) according to the manufacturer's protocol [16, 33], and the antibiotics-resistant clones were selected with the culture media containing 0.5 mg/ml Geneticin^®^. A549 and SBC5 cells were transfected with SUV39H2-specific siRNA duplex, LSD1-specific siRNA duplex or siEGFP siRNA duplex as a negative control, respectively, by using Lipofectamin RNAiMAX (Life Technologies, Carlsbad, CA) according to the manufacturer's recommendations. The siRNA sequences are described in Supplementary Table S2.

Mouse *Suv39h2* wild-type and *Suv39h2*^−/−^ MEF cells were established by Dr. Thomas Jenuwein group [21, 23]. Cells were cultured with Dulbecco's modified Eagle's medium (D-MEM) supplemented with 10% fetal bovine serum, 1% antibiotic/antimycotic solution (Sigma-Aldrich, St. Louis, MO), 2 mM L-glutamine, 0.1 mM β-mercaptoethanol, 1x non-essential amino acid solution (Life Technologies) and sodium pyruvate.

### Mass spectrometry

The reaction mixture of *in vitro* methyltransferase assay was subjected to SDS-PAGE, and the bands on the gel were visualized by SimplyBlue^TM^ SafeStain (Life Technologies, Carlsbad, CA). The bands corresponding to LSD1 were excised from the gel, and digested with sequencing grade TPCK-trypsin (Worthington Biochemical, Lakewood, NJ) in 30 μL of digestion buffer (10 mM Tris-HCl, 0.05% decyl glucoside, pH 8.0) at 37°C for 12 h. The digest mixture was separated using a nanoflow LC (Easy nLC, Thermo Fisher Scientific, Waltham, MA) on an NTCC analytical column (C18, Φ0.075 × 100 mm, 3 μm, Nikkyo Technos, Tokyo, Japan) with a linear gradient of 35% buffer B (100% acetonitrile and 0.1% formic acid) at a flow rate of 300 nL/min over 10 min, and subjected on-line to a Q-Exactive mass spectrometer (Thermo Fisher Scientific) with a nanospray ion source using data dependent TOP10 method. The MS/MS spectra were searched against the in-house database using local MASCOT server (version 2.3; Matrix Sciences, London, United Kingdom). The quantitative analysis using Qual Browser (version2.2; Thermo Fisher Scientific) was performed as described previously [7].

### RNA extraction, real-time PCR

Total RNA is purified from the cells by using RNeasy Mini Kits (QIAGEN, Venlo, Netherlands) according to manufacturer's recommendations. Synthesis of cDNA is performed with SuperScript^TM^ III First-Strand Synthesis System for RT-PCR kit (Life Technologies). Detailed information for the primers is available in Supplementary Table S3.

### *In vitro* methyltransferase assay

*In vitro* methyltransferase assays were performed as described previously [7, 44-50]. Briefly, 1 μg of His-LSD1 protein was incubated with 1 μg of His-SUV39H2 in 50 mM Tris-HCl (pH 8.8), 1.0 μCi/ml S-adenosyl-L-[methyl-^3^H]-methionine (Perkin Elmer, Waltham, MA) and Milli-Q water for 1 hour at 30°C. After boiling in sample buffer, the samples were subjected to SDS-PAGE, and visualized by fluorography [44].

### Immunoprecipitation and antibodies

293T cells were seeded at a density of 40% on a 100-mm dish. After cell attachment, the cells were transfected with expression vectors using FuGENE6, and after 48 h, transfected 293T cells were washed with PBS and lysed in RIPA buffer (50 mM Tris-HCl (pH 7.4), 150 mM NaCl, 0.5% sodium deoxycholate, 0.1% SDS, 1% Nonidet-P40, 0.1 mM PMSF) with complete protease inhibitor cocktail (Roche Applied Science). Cell extracts were incubated with anti-FLAG M2 agarose (Sigma-Aldrich) for 2 h at 4°C. After the beads were washed 3 times with 1 ml of TBS buffer (pH 7.6), the FLAG-tagged proteins bound to the beads were eluted by boiling in Lane Marker Sample Buffer (Thermo Fisher Scientific). Samples were then subjected to SDS-PAGE, and detected by western blot. The following antibodies were used: anti-LSD1 (ab17721, Abcam, Cambridge, UK), anti-CoREST (ab32631, Abcam), anti-HDAC1 (sc7872, Santa Cruz Biotechnology, Dallas, TX), anti-HDAC2 (sc7899, Santa Cruz Biotechnology), anti-Ubiquitin (sc8017, Santa Cruz Biotechnology), anti-H3K4me1 (ab8895, Abcam), anti-H3K4me2 (ab32356, Abcam) and anti-histone H3 (ab1791, Abcam).

### *In vivo* labeling

*In vivo* labeling was performed as described previously [51]. 293T cells were starved for 0.5 hour in methionine-free medium, including cycloheximide (100 μg/ml) and chloramphenicol (40 μg/ml). They were then labeled with L-[methyl-^3^H] methionine (10 μCi/ml, Perkin Elmer) for 3 hours. FLAG-LSD1-WT or FLAG-LSD1-K322R in the presence of HA-SUV39H2 was immunoprecipitated with FLAG-M2 agarose and methylated LSD1 was visualized by fluorography.

## SUPPLEMENTARY MATERIAL FIGURES AND TABLES


