# Supplementary material for: SUV39H2 methylates and stabilizes LSD1 by inhibiting polyubiquitination in human cancer cells
Source: Oncotarget. 2015 Jul 3;6(19):16939–50. doi: 10.18632/oncotarget.4760 (PMC4627283; doi:10.18632/oncotarget.4760)
Supplement: Supplementary file 1 [file oncotarget-06-16939-s001.pdf]

SUV39H2 methylates and stabilizes LSD1 by inhibiting polyubiquitination in human cancer cells

Supplementary Material

The file contains

Supplementary Information Figure S1 - S2

Supplementary Information Tables S1 - S3

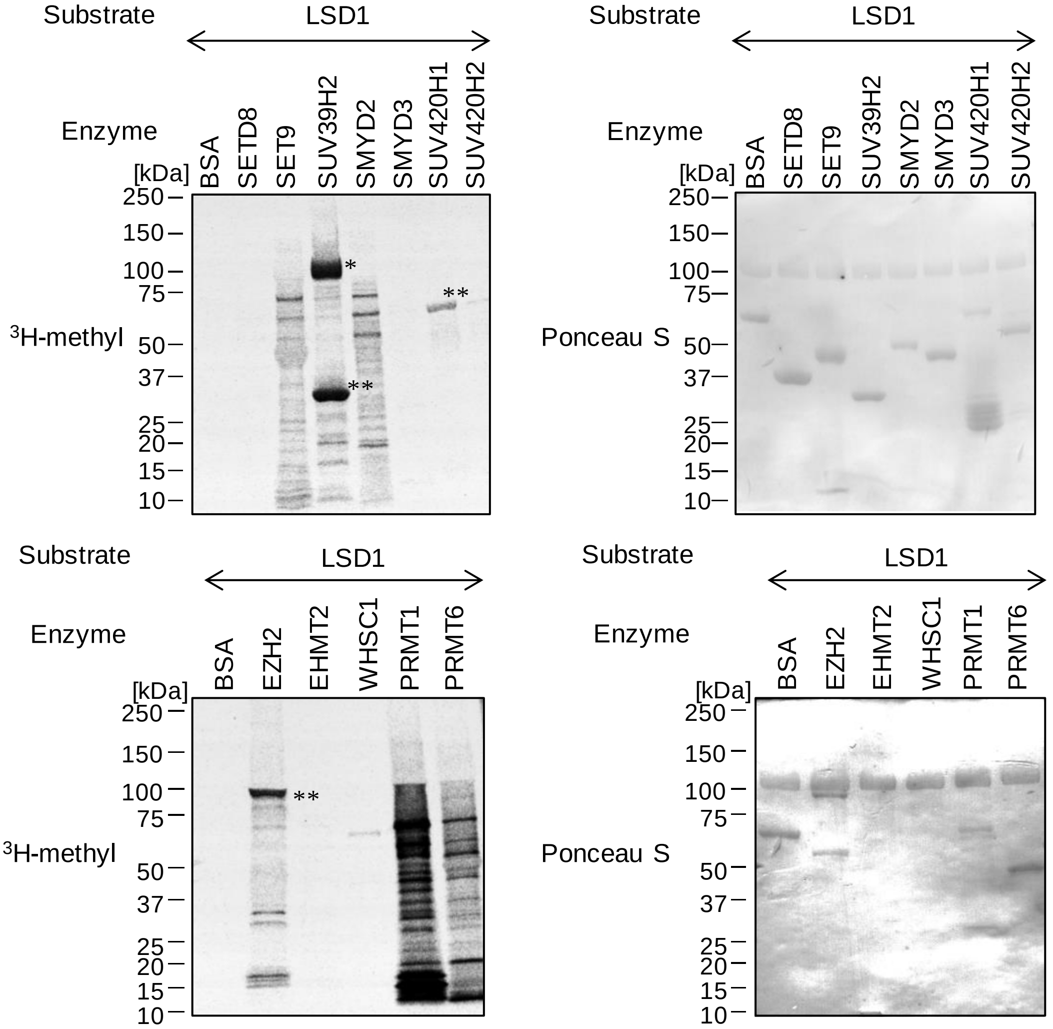

Figure S1: SUV39H2 methylates LSD1 *in vitro*. Recombinant LSD1 protein was incubated with a variety of methyltransferases, and methylaton signal was detected by autoradiography. Amounts of loading proteins were evaluated by staining with Ponceau S. *, LSD1 methylation band. **, automethylation band.

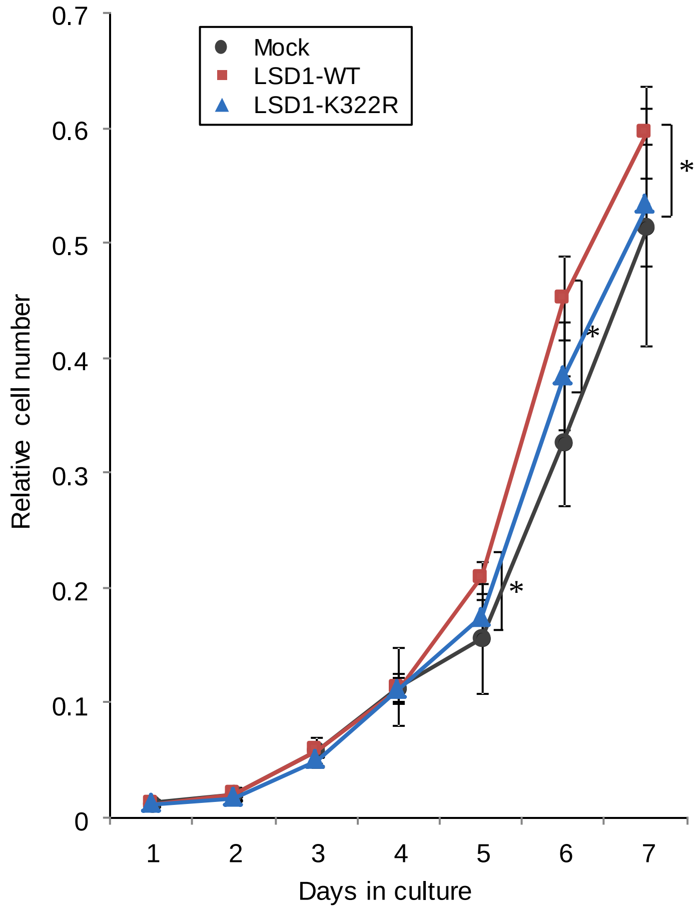

Figure S2: The cell growth assay of stable transformants. HeLa cells were transfected with Mock, LSD1-WT and LSD1-K322R expression vectors and selected by geneticin (G418) as described in Methods. Relative cell amount was measured by cell counting kit 8 (CCK8): results are the mean ±SD of three independent experiments. *P* values were calculated using Student's *t*-test (*, $P < 0.05$).

Table S1: Information of certificated cell lines

Name	Certification institution	Tested method	DNA profile
**293T**	ATCC	STR	Amelogenin: X CSF1PO: 11, 12 D13S317: 12, 14 D16S539: 9, 13 D5S818: 8, 9 D7S820: 11 THO1: 7, 9.3 TPOX: 11 vWA: 16, 18, 19
**HeLa**	ATCC	STR	Amelogenin: X,Y CSF1PO: 11,12 D13S317: 11,14 D16S539: 9,11 D5S818: 11,12 D7S820: 10,11 THO1: 8 TPOX: 8 vWA: 15
**A549**	ATCC	STR	Amelogenin: X,Y CSF1PO: 10,12 D13S317: 11 D16S539: 11,12 D5S818: 11 D7S820: 8,11 THO1: 8,9.3 TPOX: 8,11 vWA: 14
**SBC5**	JCRB	STR	Amelogenin:XY TPOX:9,12 CSF1PO:10 D5S818:10,11 D13S317:8,10 D7S820:8,11 D16S539:12 vWA:14,18 TH01:6

ATCC; American Type Culture Collection

JCRB; Japanese Collection of Research Bioresources

Table S2: siRNA sequences

siRNA name	Sequence	
siEGFP	Sense	GCAGCACGACUUCUUCAAG
	Antisense	CUUGAAGAAGUCGUGCUGC
siSUV39H2#1	Sense	CACAGAUUGCUUCUUUCAA
	Antisense	UUGAAAGAAGCAAUCUGUG
siSUV39H2#2	Sense	CUGGAAUCAGCUUAGUCAA
	Antisense	UUGACUAAGCUGAUUCCAG
siLSD1	Sense	CUAUGUAGCUGAUCUUGGA
	Antisense	UCCAAGAUCAGCUACAUAG

Table S3: Primer sequences for quantitative RT-PCR

Gene name	Primer sequence
hSOX2 - f	5’ TGCGAGCGCTGCACAT 3’
hSOX2 - r	5’ TCATGAGCGTCTTGGTTTTCC 3’
hCoREST - f	5’ CCGCCAAACTGGCAAGAC 3’
hCoREST - r	5’ TGGGTGACCAGACCAACATG 3’
hLSD1 - f	5’ TGGTGGTAACAGGTCTTGGAGG 3’
hLSD1 - r	5’ GGCTTCATAAAGTGGGCATTTTTG 3’
hSUV39H2 - f	5’ TGGGGTGTAAAGACCCTTGTG 3’
hSUV39H2 - r	5’ ATTCCCTTGTTGTCATAGAAC 3’
hCDKN1A - f	5’ GGAAGACCATGTGGACCTGT 3’
hCDKN1A - r	5’ GGCGTTTGGAGTGGTAGAAA 3’
mCdkn1a - f	5’ TTGCACTCTGGTGTCTGAGC 3’
mCdkn1a - r	5’ TCTGCGCT GGAGTGATAGA 3’
mSox2- f	5’ GAACGCCTTCATGGTATGGT 3’
mSox2- r	5’ TTGCTGATCTCCGAGTTGTG 3’
